# HybriD-GM: A Framework for Quantum Computing Simulation Targeted to Hybrid Parallel Architectures

**DOI:** 10.3390/e25030503

**Published:** 2023-03-14

**Authors:** Anderson Avila, Helida Santos, Anderson Cruz, Samuel Xavier-de-Souza, Giancarlo Lucca, Bruno Moura, Adenauer Yamin, Renata Reiser

**Affiliations:** 1 Center of Technological Development, Federal University of Pelotas, Pelotas 96010-610, Brazil; 2 Centro de Ciências Computacionais, Universidade Federal do Rio Grande, Rio Grande 96201-900, Brazil; helida@furg.br (H.S.);; 3 Institute of Smart Cities, Universidad Pública de Navarra, 31006 Pamplona, Spain; 4 Navarra Artificial Intelligence Research (NAIR) Center, 31006 Pamplona, Spain; 5 Metropole Digital Institute, Federal University of Rio Grande do Norte, Natal 59072-970, Brazil; 6 Department of Computer Engineering and Automation, Federal University of Rio Grande do Norte, Natal 59078-970, Brazil; 7 Diretoria de Tecnologia da Informação e Comunicação, Universidade Federal do Pampa, Bagé 96400-100, Brazil

**Keywords:** quantum computing, hybrid computing, Shor’s algorithm, Grover’s algorithm, quantum simulation

## Abstract

This paper presents the HybriD-GM model conception, from modeling to consolidation. The D-GM environment is also extended, providing efficient parallel executions for quantum computing simulations, targeted to hybrid architectures considering the CPU and GPU integration. By managing projection operators over quantum structures, and exploring coalescing memory access patterns, the HybriD-GM model enables granularity control, optimizing hardware resources in distributed computations organized as tree data structures. In the HybriD-GM evaluation, simulations of Shor’s and Grover’s algorithms achieve significant performance improvements in comparison to the previous D-GM version, and also with other related works, for example, LIQUi|⟩ and ProjectQ simulators.

## 1. Introduction

Quantum computing (QC) promises to solve some problems that in classical computing would be impractical. QC systems and near-term quantum techniques have raised interest over the last decades [1,2]. Thus, the imminence of quantum supremacy is a reality, meaning that a quantum computer can perform a calculation task that would be intractable even by a supercomputer using the usual programming paradigms [3,4].

Several quantum algorithms (ordering, prime factorization, and modular exponentiation, among others) have already been developed, leading to significant improvements when compared to the best-known classical algorithms. Consequently, quantum computing has been considered potentially significant in many areas such as reversible logic, quantum cryptography, information processing, communication, and data coding methods [5].

However, quantum computers are still in their early days, requiring specialized and expensive infrastructure. Although quantum hardware’s construction prevails as a technological challenge restricted to laboratories and huge companies, it might become a feasible technology in the near future.Until quantum computers become widely available, the development and testing of quantum algorithms may be done by simulating quantum computing on classical computers. Although specific classes of quantum algorithms can be efficiently simulated [6,7,8,9,10], this is not the case for most quantum algorithms, as the representations of quantum states (QSs) and quantum transformations (QTs) grow exponentially [11].

This research promotes a simulation of general quantum algorithms by classical technologies, mainly motivated by the following arguments.

(i) Although the construction of quantum hardware is still a relevant issue, existing quantum algorithms present significant improvements compared to the best-known classical algorithms [5].
(ii) Better understanding the many classes of quantum algorithms and their behaviors might be the first step toward an approach to resolve huge computational problems in emerging computing areas, such as artificial intelligence and robotics, big data analysis, bioinformatics, and cyber security [12].
(iii) The progress on new computation techniques allows to simulate quantum algorithms, integrating relevant areas, ranging from cryptography to cosmology and quantum neural computing to quantum chemistry. It appears as a new paradigm built upon the combination of artificial intelligence and neural computation. Simulations modeling brain functionality and interpreting the behavior of a quantum algorithm (QA) have contributed to creating new systems for information processing, including new solutions for classically intractable problems [13].
(iv) Simulations of a generic QA on classical computers is a demanding task both regarding temporal and spatial complexity. As quantum states may be represented as vectors and quantum transformations, as matrices, the register sizes increase exponentially with the number of qubits of the application [14,15].
(v) New methodologies dealing with quantum computational models are grounded on understanding the power and complexity of QC by resorting to the known capacity of learning about the main characteristic of good results of quantum algorithms.
(vi) Until quantum computers become broadly available, the development and testing of QAs may be done by simulation, providing many advantages over analytical processes, such as the detailed study of their behavior without supporting a quantum physical environment.

Therefore, the major objective of this work is to develop the HybriD-GM model, conceived as a computational methodology for quantum computing simulations and targeted to the classical hybrid architecture, in order to better assist the study of quantum algorithms. The HybriD-GM model explores high-performance computing (HPC) potentialities to improve performance by (i) optimizing resources and enabling hardware-independent scalability; and (ii) exploring composition and projection operators, also including coalescing memory management. Both strategies act on quantum structures in order to obtain a hybrid structure integrating CPU and/or GPU architectures.

Moreover, the HybriD-GM also promotes an effective extension of the D-GM framework, which has been under development by our research group, in LUPS at UFPEL, see [16,17,18,19]. This fact supports the HybriD-GM validation process carried out in testing, which achieved significant performance improvements over its previous version (D-GM) and the LIQUi|〉 and ProjectQ simulators, as it will be presented in this paper.

The remaining sections of the paper are organized as follows. Section 2 discusses the related works, including the D-GM approach. Section 3 presents the conceptual model of the HybriD-GM approach, and in Section 4, we have the architectural model. In Section 5, we characterize the execution approaches considered in this work, which enables the execution of simulations. In Section 6, the structure of the GPU kernel for evaluating applications is addressed, including Section 6.1 and Section 6.2, where we have the results concerning only CPU evaluation applications and only GPU evaluation applications, respectively, and Section 6.3, discussing the results when performing a hybrid simulation in CPU and GPU. At last, Section 7 promotes a general discussion on the results of the HybriD-GM model, with comparisons between our proposal and other simulators.

## 2. Related Works

Currently, many approaches on QC simulation can be found in the literature. We searched related works which could be compared to our proposal, according to the following features:

(i) Consolidated projects dealing with general-purpose quantum computing simulation;
(ii) HPC approach having multi-core CPU, GPU, and/or distributed executions;
(iii) Available reports from main characterizations, optimizations, and simulation results.

The result was six selected works: LIQUi|〉 [20], *q*H*i*PSTER [21], ProjectQ [22], Haner [23], Gutierrez [24] and Zhang [25], whose detailed analysis and summarized comparisons were studied. We realized that none of the simulators has a hybrid approach combining CPU and GPU. Additionally, despite such interesting optimization strategies, they are targeted to a specific architecture.

Overcoming the above restriction, this work was conceived considering a computational model for QC simulation, which can be applied to general-purpose algorithms, optimizing its resources as well as allowing hybrid simulations.

Next, a brief analysis of the six selected quantum simulators (S1–S6) is presented, considering the multicore architectures’ application, single or multi-GPU exploration, the distribution of computations, and circuit optimizations.

(S1) LIQUi|〉 simulator has its mains computation running in a functional language (F#) [20], and is presented as one of the best options for high-performance applications. Two interesting optimizations should be emphasized: (i) gate growth, allowing to reduce significantly the number of gates on a QA; and (ii) full rewrite of the complex math package, drastically reducing simulation time.
(S2) *q*H*i*PSTER presents results to understand its performance behavior on a supercomputer but restricted to small cases, as complex algorithms were not simulated, see more details in [21]. A multi-qubit simulation, over 29 qubits of a quantum Fourier transformation (QFT) in a single node, took 116.6 s while the simulation on the D-GM approach took less than 5 s. The simulation time remains very large, independently of the selected (double-/single-) precision storing the state vector.
(S3) ProjectQ seems interesting as an open-source framework, but the work seen in [22] does not present any detailed information about its simulator for general QA. Despite presenting better results than LIQUi|〉 and *q*H*i*PSTER, a simple hardware (notebook with only two cores) was used on the simulations. Further investigations are needed to evaluate its performance over robust parallel processing power architectures.
(S4) Haner’s simulator applies all the optimizations on the table for multi-core and distributed simulations; refer to [23] for further details. Since it was built to primarily simulate quantum supremacy algorithms, the main optimizations and presented results are targeted toward these algorithm types, making an analysis performed over more complex algorithms difficult.
(S5) Gutierrez’s simulator is the oldest selected simulator, being introduced in 2010 [24]. Since then, despite GPU having further increased both performance and memory storage, its strategy shows an approach similar to the D-GM model, considering coalesced access to GPU global memory. However, it is not possible to directly compare results with newer hardware.
(S6) Zhang’s simulator, presented in [25], considers a single node multi-GPU implementation. It has a kernel similar to [24] and uses the same approach presented in [23] to avoid communication between devices and reduce memory transfers. The results presented consider only QFT and a comparison with intermediate versions of itself. A performance scalability analysis is not discussed.

The D-GM framework, in turn, is able to simulate QAs, and it provides graphical interfaces for modeling applications either sequentially or in parallel, using either multi-core CPUs or multi-core GPUs. The D-GM model is detailed in [19], and its sources are available at a public repository (github.com/abdavila/D-GM-QC-Simulator). Such a previous approach is structured as follows:

***Quantum circuit level:*** describes the application in the circuit model and then automatically exports it to a representation for the qGM model;
***qGM level:*** contains the visual programming environment for the qGM model (VPE-qGM), which allows the user to describe/simulate computations under the qGM.
***D-GM level:*** implements the distributed simulation manager, Virtual Distributed Geometric Machine (VirD-GM), which handles tasks such as scheduling, communication, and synchronization required in distributed simulations.
***Hardware level:*** enlists all the devices that can be used by the framework, from regular desktops for sequential simulations to clusters with multiple GPUs.

## 3. HybriD-GM Conceptual Model

The state space and operators in QC are mathematically described by the Hilbert space. A quantum register comprising a number of qubits is given as a vector in a multidimensional Hilbert space. In addition, quantum gates are Hilbert space operators that rotate the quantum register vectors [26].

### 3.1. Computations with Projections over Quantum States

**Figure 1:**
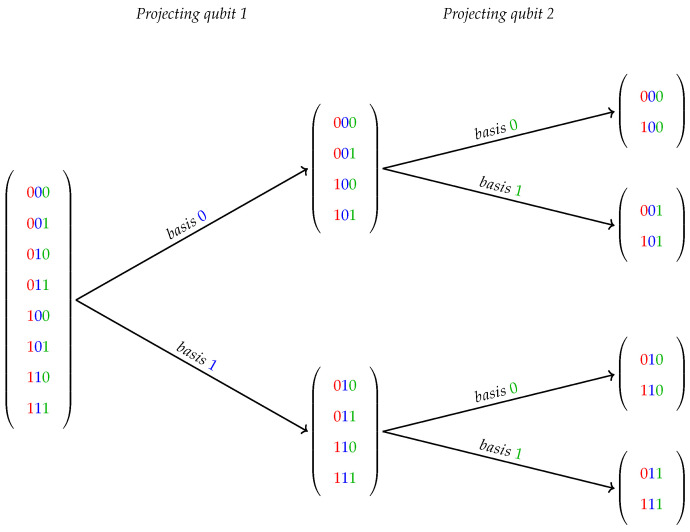
Projections of first and second qubits in a 3-dimensional quantum state.

**Figure 2:**
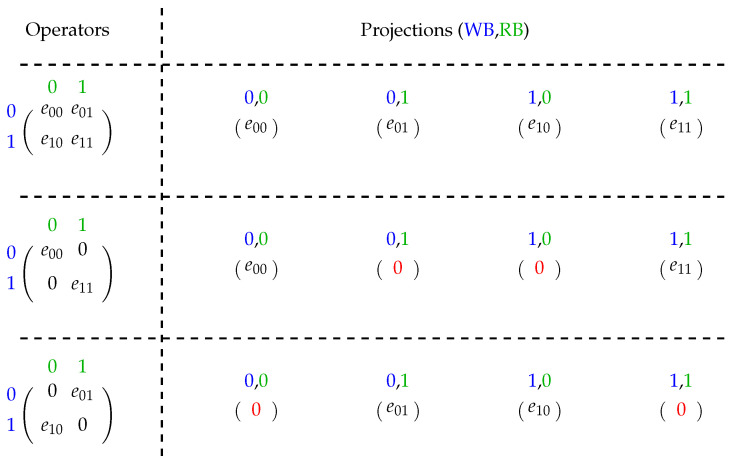
Projections of quantum operators.

The HybriD-GM model explores not only the intrinsic characteristics of projection operators usually applied to quantum states but also the dynamic of computations based on projected structures. Three of the main strategies are detailed as follows:

(I) As a projection applied to a QS structure implies on partitions of its classical basis components, it results in subsets containing their corresponding amplitudes. See, for instance, Figure 1 showing a generic 3-qubit state, where amplitudes represented by their binary position are projected on its second and third bases over a two-step projection process.

(II) Regarding basis descriptions for memory values applied in a projection operator:
  **WB**: indicated by matrix lines whenever the write basis value is associated with the quantum state basis it computes;
  **RB**: indicated by matrix columns, where the RB value is related to the quantum state basis it uses for the computation.
  Thus, single-qubit quantum operators can be classified into three types according to their non-zero values as shown in Figure 2, generating four pair-projections of combinations between the WB and RB basis. Therefore, for dense operators, each WB is associated with two RBs. For sparse ones, each WB is associated with only one RB.

(III) By optimizing the tensor product, a QT projection for a given basis can be obtained by individually projecting the operator related to that basis and, only then, performing the multiplication of the projection values with the tensor product between the other operators, for instance, in a 2-dimensional QT, given by Id⊗H and related to projection over the first basis which is related to the Id-operator. Thus, see the results in the projections on the first basis of the quantum transformation Id⊗H:
  $$\begin{array}{cccc} 0,0 & 0,1 & 1,0 & 1,1\\ \left(1\right) & \left(0\right) & \left(0\right) & \left(1\right) \end{array}$$
  Therefore, each value applied to the *H*-operator results in the following matrices:
  $$\begin{array}{cccc} 0,0 & 0,1 & 1,0 & 1,1\\ \left(\begin{array}{cc} \frac{\sqrt{2}}{2} & \frac{\sqrt{2}}{2}\\ \frac{\sqrt{2}}{2} & -\frac{\sqrt{2}}{2} \end{array}\right) & \left(\begin{array}{cc} 0 & 0\\ 0 & 0 \end{array}\right) & \left(\begin{array}{cc} 0 & 0\\ 0 & 0 \end{array}\right) & \left(\begin{array}{cc} \frac{\sqrt{2}}{2} & \frac{\sqrt{2}}{2}\\ \frac{\sqrt{2}}{2} & -\frac{\sqrt{2}}{2} \end{array}\right) \end{array}$$

### 3.2. Computations with Projections over Matrix Structures

Given a QS and a QT projected on qubits/operators (over related basis), the application of a QT on this QS computes the multiplication between each QT projection and the correspondent QS projection (defining an RB value) and then, the sum of results associated to a WB is executed, obtaining the QS resultant for such a basis.

**Figure 3:**
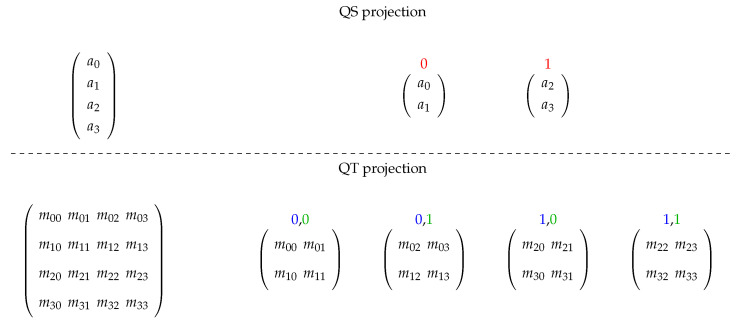
Structure projection for a generic 2-qubit system.

**Figure 4:**
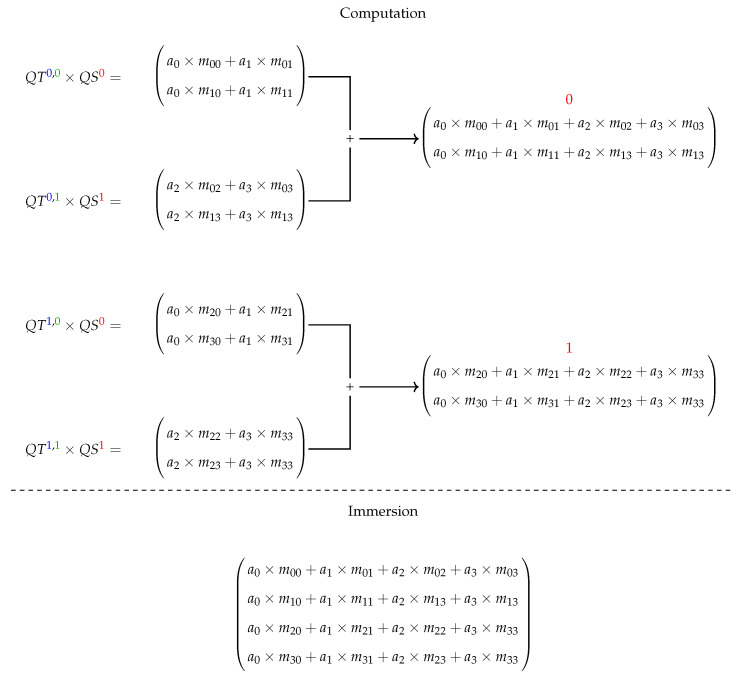
Computation of a generic 2-qubit system.

An example of a generic 2-qubit system having the first qubit/operator projected is presented in Figure 3 and Figure 4, showing the projections acting on computations with matrix structures. First, in Figure 3, we see how the structures are projected, and then immersion operators execute the computations followed by the reconstruction of the quantum state in Figure 4.

## 4. HybriD-GM Architectural Model

The HybriD-GM model explores the projection of quantum states and transformations to control the distribution and granularity control of distributed computations while optimizing hardware resources. This proposal targets hybrid architectures handling the CPU/GPU of single machines, allowing extensions to other architectures due to their flexible structure. HybriD-GM data structures have three levels:

(1) **Projection structure**, containing QS amplitudes that can be stored directly or indirectly, and all information necessary for access and manipulation.
(2) **Projection instance**, providing a reference to the generated projection structure, enumerating the projected basis values.
(3) **Gate structure**, containing the matrix construction of QT, including information on the target qubit and control qubits/values, if any.

**Figure 5:**
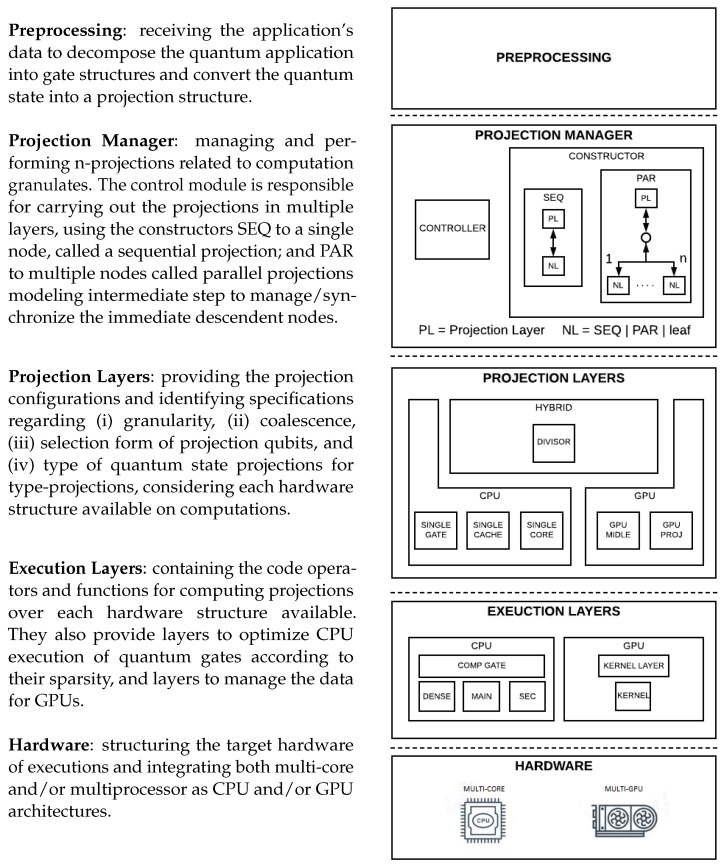
HybriD-GM architecture.

In Figure 5, the architectural structure of the HybriD-GM model is graphically presented in the sequence.

Now, let us see each of these levels in more detail.

### 4.1. Characterizing the Projection Manager Structures

The projection manager of the HybriD-GM model manages and performs the projections according to the quantum application. The control module is responsible for carrying out the projections in multiple layers and controlling the granularity of the computations as well as optimizing the hardware resources. Next, we present four methodologies within the projection manager, including (i) the project manager, (ii) the projection qubits selection, (iii) the project quantum state, and (iv) the project quantum transformation.

First, we use main builders in the *project manager* to define the graph data structure, which is conceived as a tree describing projections/computation considering SEQ and PAR operators, where SEQ (to a single node) is a sequential projection and PAR (to multiple nodes) performs parallel projections, modeling an intermediate step to manage/synchronize the immediate descendant nodes.

The nodes presented in those builders have the following definition:

- PL—projection layer, which is conceived as projection nodes (intermediate-nodes);
- NL—next (projection) layer, which can be one of the two builder types (SEQ and PAR), and adding another projection layer, as well as an execution node (leaf-node) ending the branch of a projection tree.

Other main nodes present the following features:

- **Node structure** receives a projection structure and a list of gate structures to be projected or computed;
- **Root node** receives the structures created in the preprocessing level;
- **Children nodes** have a smaller granularity than their parent node, meaning that the lower the level in a branch-tree, the smaller the projection size they will work on;
- **Sibling nodes**, from the same parent node, will have the same granularity, but adjacent nodes belonging to another parent may have another.

**Figure 6:**
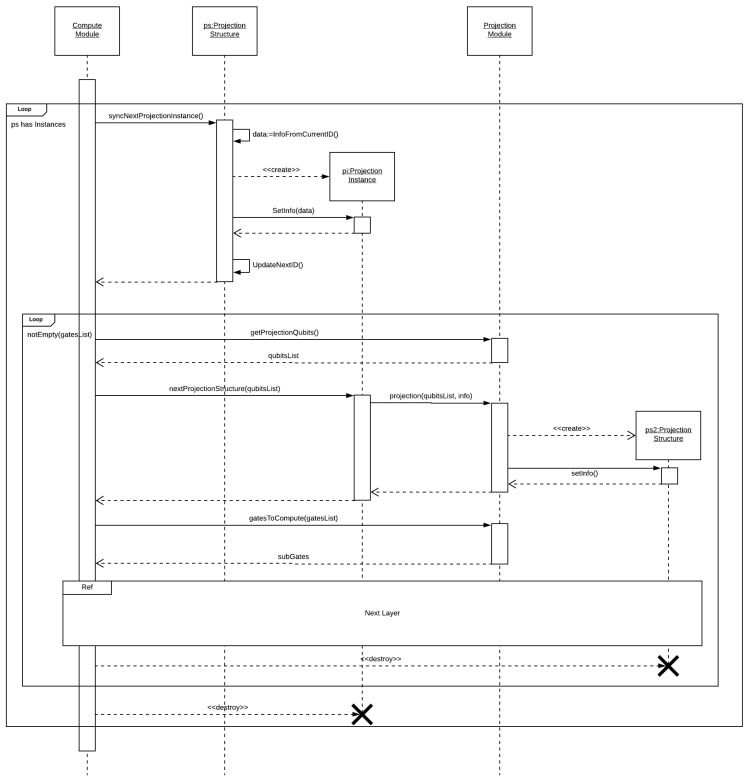
Generic projection layer.

In Figure 6, we see the flow diagram for a projection node, which receives a projection structure and a list of gate structures. The control module performs a main loop requesting projection instances from the projection structure. For each projection instance, a secondary loop is executed until all gate structures have been computed. On each iteration, we use the projection instance and the configurations associated with the current layer to generate a set of qubits (basis) to be projected. Using this set of qubits, two structures are generated, namely (i) a new projection structure, with a lower granularity, and (ii) a sub-list of the gate structure, with the gate structures related to the new projection structure.

Thus, these structures are passed to the NL (next layer) awaiting the return to move on to the next iteration. After all projection instances have been computed, the parent node is notified, concluding the control module execution.

Next, we describe the *projection qubits selection* methodology. Consider *m* the number of qubits to be projected and *n* the number of qubits of the quantum state. The selection of the projected qubits can be performed using distinct strategies:

(1) **Interval data**, where the selected projection qubits are *m*-dimensional intervals considering two options: (i) *Static structured* is selected from a previously defined set of intervals that together cover all *n* qubits and, therefore, ensure that all quantum gates will be associated to at least one interval. For example, take the following intervals: [0;m−1],[m;2m−1],…,[n−m:n−1]. These can be selected in an alternate manner and repeated until all operators have been computed; (ii) *Adaptable structured*, where the information of the next operators in the list of gate structures is used to define the interval, by adding their target qubits to a set until the interval between the smallest and the largest number does not exceed the limit of *m* qubits.
(2) **Dynamic data**, where the set of qubits can be defined dynamically, allowing only qubits with operators to be chosen for the next projection step. The non-executed operators can be iterated, and non-duplicated target qubits are added to a set until its size reaches *m*.
(3) **Fixed data**, where projections are always performed over predefined set of *c* qubits, with c<m. This implies the need for a combination with one of the previous approaches to cover the total selection of *m* qubits. After disregarding those *c* qubits as selection options, the other approaches can be performed to choose the remaining m−c qubits. Fixing the first *c*-qubits of a quantum state implies that all projection instances will have contiguous segments with a minimum of $2^{c}$ amplitudes.

In the third methodology of the projection manager structure, the *project quantum state*, we describe two types of projections (direct and indirect), regarding the quantum states on projection structures:

(1) **Direct projection**, where amplitudes corresponding to a given projection instance are copied/transferred to a new memory space. Therefore, information from previous projections does not have to be carried out for further projections/computations to be performed, and, consequently, it can be interpreted as a root projection. Despite being mainly related to projections from one memory architecture to another, nothing prevents it to be applied in the same architecture. In the context of this work, it is used to project from CPU RAM to GPU RAM, and from GPU RAM to GPU shared memory. However, it is not limited to those since it can also be used to perform projections of HARD DISK to CPU RAM, or between nodes in a cluster, for instance.
(2) **Indirect projection**, where the projection state containing the projection instance is passed as a reference, implying the use of information regarding the projection instance to determine the access of its amplitudes. When performing successive indirect projections, it is necessary to have the combined information of previous projections in the branch tree up to the closest that can be interpreted as a root projection. It is mainly related to projections in the same memory architecture, preventing intermediate copies of the projections from being unnecessarily performed.

Finally, in the *project quantum transformation* methodology, two main characteristics should be highlighted:

- **Constructing the gate structured list**: after defining the projection qubits, operators acting on non-projected qubits are transferred to another list, as long as they can be executed preserving the computation consistency.
- **Passing data by reference**: this can be applied if the state projection performed was indirect since the acting and control qubits remain the same. Otherwise, it must copy/transfer the operator and map their target and control quits to match their correspondents in the new quantum state. For the node independence related to previous projection information in tree-branch modeling, the application is preserved.

Next, we provide an example with a quantum algorithm with six qubits and five quantum transformations.

**Figure 7:**
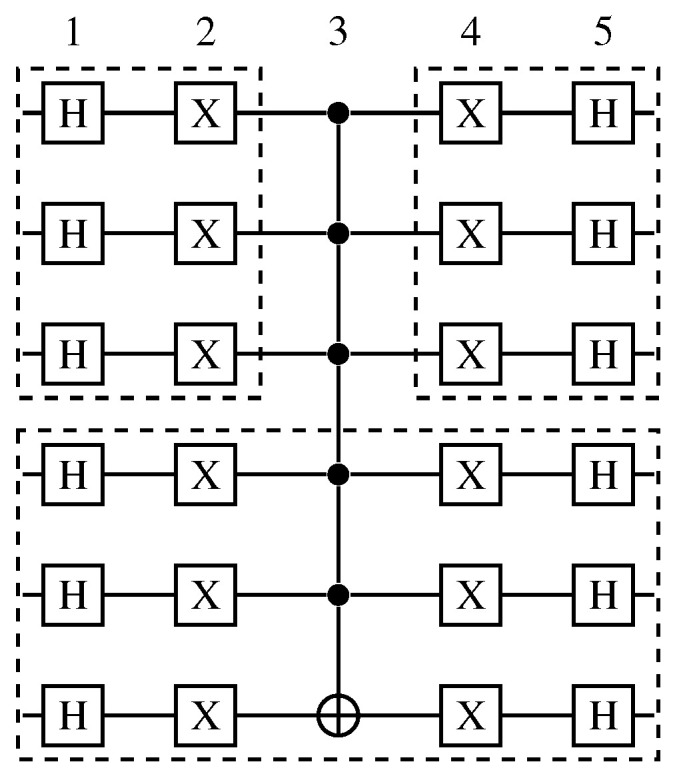
Diffusion operator with 6-qubits.

**Example 1.**  *Take the 6-qubit QA in Figure 7 containing 5 QTs performed over 6 QSs. Define two sets of qubit-projections [0–2],[3–5]. Observe that the only dependency present in the QA is the controlled transformation in the third QT. So, every quantum gate on QT 1–2 has to be executed before the operators in QT 4–5 to maintain the result consistency. So, the best projection sequence would be to first project [0–2] and execute the gates in QT 1–2 for that interval, allowing to project [3–5], and then execute all gates in this interval to finally project [0–2] and execute the remaining gates in QT 4–5.*

*Note that optimizations on quantum transformation projections were not included in the current state of the HybriD-GM model. So, instead of having three execution steps in this example, eight steps would be necessary to perform the same QA.*

### 4.2. Characterizing the Projection Layer Structures

The projection layers level of the HybriD-GM model contemplates three categories of projections in the context of hybrid architectures, related to the type of simulation: CPU, GPU, and hybrid approaches. The proposal targets granularity, coalescence, the selection form of projection qubits, and the type of quantum state projections to optimize hardware resources for various scenarios, whose requirements are described as follows.

1 CPU projection requirements for CPU simulations:

**Single gate**, considering 1-level granularity and no coalescence, the target qubit of the first operator in the list of gate structures is used to determine the projection qubit, and this operator is also individually projected.
**Single cache**, determined by granularity and coalescence associated to the projected quantum state, with respect to the memory space and its chunks of subsequent amplitudes. It cannot exceed the sizes of the last cache level to each core and the first cache level, respectively. It uses fixed and dynamic approaches to select the projection qubits.
**Single core**, where the granularity and coalescence are always greater values than the ones for single cache, with values depending on the simulation type.

2 GPU projection requirements for GPU simulations:

**GPU MIDDLE**, granularity limited to not exceed the GPU memory size, and coalescence to have the memory space of subsequent amplitudes equal or higher than the minimum memory transaction size between CPU and GPU.
**GPU PROJ**, granularity defined to not exceed the GPU shared memory per thread block, and coalescence to have chunks of subsequent amplitudes matching the transaction size between the GPU global memory and shared memory.

3 Hybrid projections allow hybrid simulations in CPU and GPU, encapsulating the projection requirements defined above, as well as the following configuration:

**DIVISOR**, where the granularity is an equal value or higher, and coalescence, an equal value to its child nodes’ maximum values.

The quantum state in the projection structure is indirectly projected for the above scenarios, except for the GPU MIDDLE category, which performs a direct projection transferring it to the GPU memory.

### 4.3. Characterizing the Execution Layer Structures

The execution layer level contemplates two execution categories, CPU and GPU.

Preliminary results performing computations using a generic projection size showed the best results on a quantum state with granularities 1 and 2. Therefore, in the CPU layer category, we considered a 1-level granularity for execution layers. This implies a gate-by-gate computation approach, allowing further optimizations according to the type of gates, which can be classified into two classes of QT:

(i) *Dense operators* defined by matrices without void elements, such as the Hadamard operator;
(ii) *Sparse operators* with void elements in the main diagonal, as the Pauli X gate, or in the secondary diagonal, such as the Pauli Y gate.

**Figure 8:**
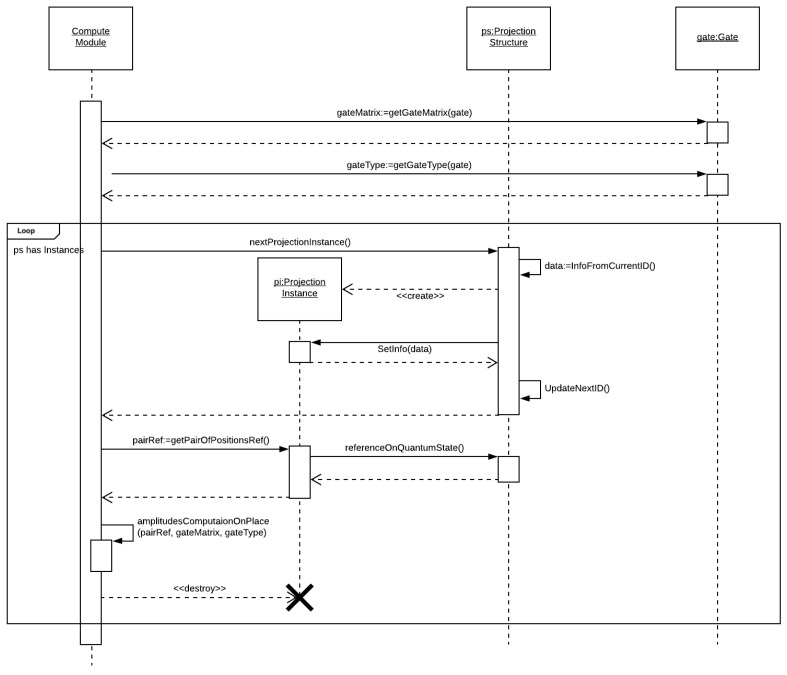
Flow diagram for the COMP GATE module.

Figure 8 illustrates the flow diagram describing the COMP GATE module, responsible for managing the computation of a single gate for a selected projection structure.

The control module requires the matrix operator and the type gate, following a loop iterating through all instances of the selected projection structure, requesting the pair of amplitudes related to the current instance. This module also invokes the corresponding computation module related to the gate type, passing the pair of amplitudes ($a_{0},a_{1}$) and the matrix (*M*).

The computation modules consider three gate types: (i) **DENSE**—for dense operators, (ii) **MAIN**—for main diagonal operators, and (iii) **SEC**—for secondary diagonal operators. The corresponding computations are described by Algorithms 1–3, respectively.

Note that the computations of the DENSE and SEC operators consider a temporary variable since the computation is performed in place and the amplitudes are used on each of the other calculations. In this case, the primary and secondary diagonal operators need to perform only two multiplications instead of four since the multiplications involving the zeros in their matrix could be removed, in accordance with the model presented in Figure 2.
**Algorithm 1:** Dense 1:$tmp\leftarrow a_{0}$ 2:$a_{0}\leftarrow a_{0}\times M\left[0\right]\left[0\right]+a_{1}\times M\left[0\right]\left[1\right]$ 3:$a_{1}\leftarrow tmp\times M\left[1\right]\left[0\right]+a_{1}\times M\left[1\right]\left[1\right]$

**Algorithm 2:** Primary Diagonal
 1:

$a_{0}\leftarrow a_{0}\times M\left[0\right]\left[0\right]$

 2:

$a_{1}\leftarrow a_{1}\times M\left[1\right]\left[1\right]$




**Algorithm 3:** Secondary Diagonal
 1:

$tmp\leftarrow a_{0}$

 2:

$a_{0}\leftarrow a_{1}\times M\left[0\right]\left[1\right]$

 3:

$a_{1}\leftarrow tmp\times M\left[1\right]\left[0\right]$




**Figure 9:**
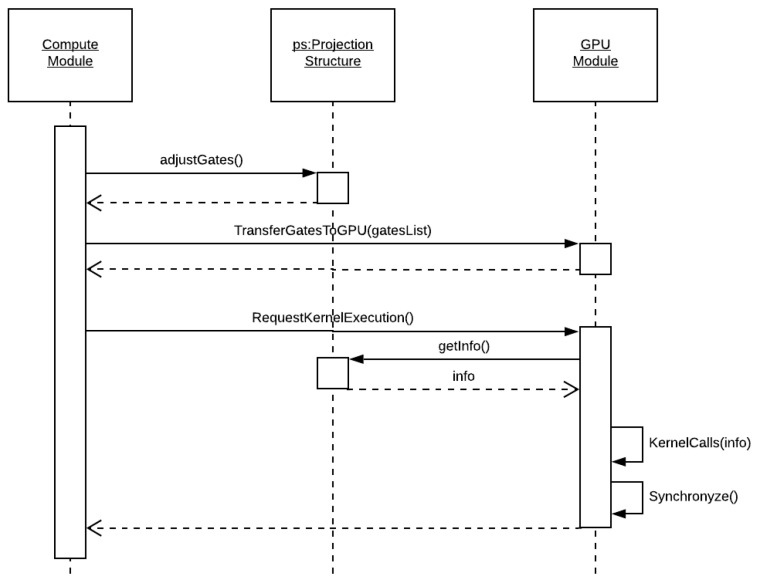
Flow diagram related to the kernel layer.

The flow diagram for the kernel layer is seen in Figure 9. This layer is responsible for requesting the GPU computation of a projection, which already has its quantum state in the GPU’s memory. The steps performed are as follows:

(i) At first, the quantum gates to be computed have their target qubits and projection controls mapped and transferred to the GPU.
(ii) In sequence, their execution is requested to the GPU module.
(iii) The GPU module retrieves the projection structure information.
(iv) At last, it invokes the GPU kernel execution on each device, waiting for their return.

In the current state of the HybriD-GM approach, multiple GPU executions consider homogeneous hardware. The execution flow for the GPU kernel is seen in Algorithm 4, considering the point of view of a GPU block, with $n_{1}$ representing the number of projection instances associated with the block.
**Algorithm 4:** Presenting flow data of a GPU kernel in a block point of view.
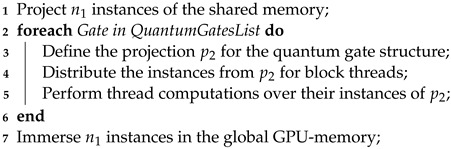


## 5. Characterizing the Execution Approaches to Allow Simulations

The projection manager of the HybriD-GM architectural model, described in Section 4, combines projection and execution layers to enable types of simulation. For all cases, the structures generated by the preprocessing steps are considered as input, meaning that a projection structure containing a single instance and a list of gate structures represent, respectively, the elected quantum state and the quantum algorithm for a simulation process.

**Figure 10:**
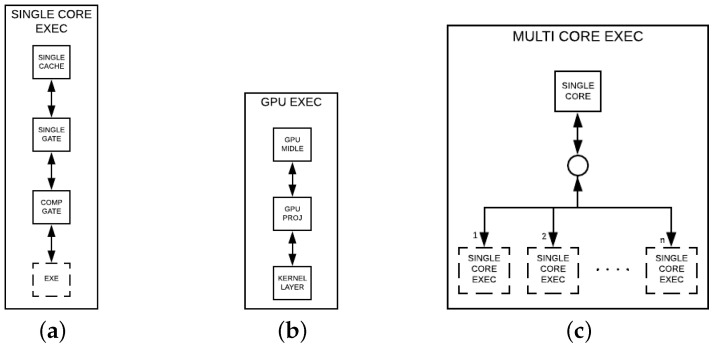
(**a**–**c**) Layer compositions for simulations.

The single core simulation in the HybriD-GM model can be observed in Figure 10a, referred to as SINGLE CORE EXEC, containing the four layers described next:

(i) SINGLE CACHE—layer ensuring that the amplitudes accessed for computing an instance will remain in the cache until the end of the computation;
(ii) SINGLE GATE—guaranteeing computations can be carried out gate-by-gate;
(iii) COMP GATE—generating ordered instances, and then ensuring the exploration of the spatial locality of the lowest cache level since each pair of amplitudes is subsequent to the previous pair;
(iv) EXE—referring to one of the following CPU execution layers, DENSE, MAIN, and SEC, which depends on the quantum gate type.

The GPU approach flow simulation seen in Figure 10b, and referred to as GPU EXEC, is performed by the following layers.

1. GPU MIDDLE—layer-generating projection structures that match the GPU memory size, and then transfer it to the GPU memory;
2. GPU PROJ—generating structures compatible with the shared memory size per GPU block;
3. KERNEL LAYER—performing kernel calls to accomplish the computation tasks.

The simulation in the multicore execution approach, in Figure 10c, takes two steps:

(i) SINGLE CORE—where the projection structures generated are passed to *n* nodes, with *n* being the number of cores used in the simulation;
(ii) SINGLE CORE EXEC—each node in this level reports to a single core simulation since it follows the same flow of projection/computations.

Multiple GPU quantum simulations follow the same approach. However, in the first layer, the quantum state from the projection structure is transferred in equal parts to each GPU, and its global memory spaces have to be visible to each other.

**Figure 11:**
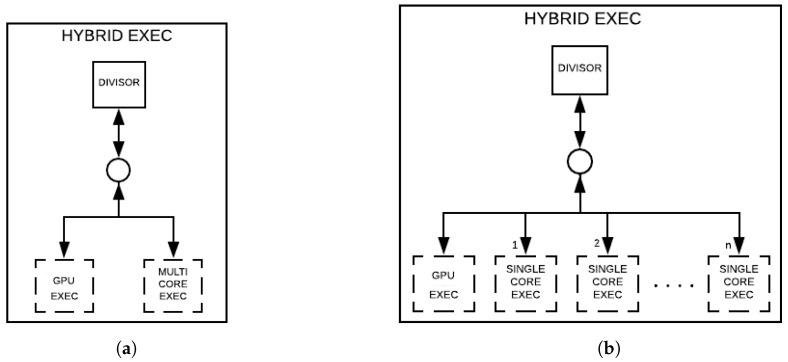
(**a**,**b**) Layer compositions for hybrid simulations.

There exist two approaches for hybrid simulations, corresponding to distinct layers, which are considered in two steps, as presented in Figure 11a,b. The first layer has the DIVISOR layer, and it is in both approaches, providing projection structures for their child nodes, which differ in each approach. The second layer is described as follows:

(i) Consisted of a pair of nodes, GPU EXEC and MULTI CORE EXEC;
(ii) Having n+1 child-nodes, GPU EXEC and *n* SINGLE CORE EXEC.

Those differences in approaches (a) and (b) will result in a distinct granularity associated with each CPU core.

## 6. HybriD-GM Proposal: Structuring GPU Kernel for Evaluating Applications

The HybriD-GM model proposed in this work can be applied to any of the simulators presented in Section 2. The current proposal is an extension of the D-GM framework [19], and the implementation was validated and assessed considering the following features.

(I) A module addition with the structures and functions present in the model that refers to the representation and manipulation of projections. All logical functions were implemented using bitwise operations to minimize their overhead.
(II) Re-implementation of computing functions in GPU and CPU following the HybriD-GM model, referring to what was modeled in Figure 10b,c, respectively.
(III) Hybrid simulation implementation combining CPU and GPU, following the model described in Figure 11b, which does not consider nested parallelism.

The CUDA (compute unified device architecture) kernel is responsible for the execution of a projection structure, preserving characteristics, functionalities, and computation methods of the HybriD-GM model. The quantum state and the gate-list structures are already in the GPU memory when the execution is requested. CUDA kernel computation may be divided into three steps.

**Step 1:** 
Identify each block projection instance, and copy their amplitudes to the shared memory. See Algorithm 5 reporting the related pseudo-code.

**Algorithm 5:** GPU Kernel Step 1.

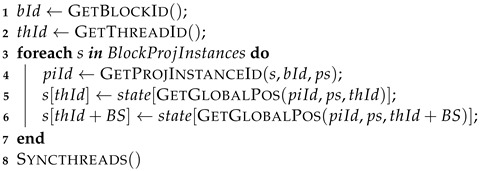

**Step 2:** 
Execute the gate-list structures. The related pseudo code is shown in Algorithm 6. This step goes through the list of quantum gates and performs their execution. In each iteration, each CUDA thread projects a pair of amplitudes according to the target qubit of the current quantum gate. Then, it calculates those positions for all sub-states (if conditions defining the quantum gate controls are satisfied). Barriers are used to synchronize these threads within the same block before passing to the next quantum gate, guaranteeing that all amplitudes on the shared memory are up-to-date.

**Algorithm 6:** GPU step 2

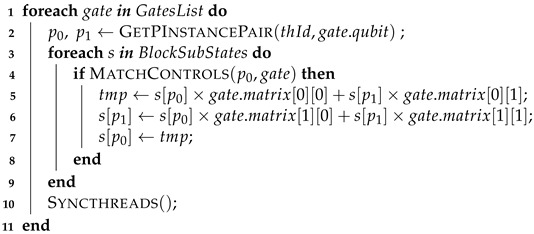

**Step 3:** 
Update the quantum state. After calculating all quantum gates, the result stored in the shared memory is copied to the quantum state on the GPU global memory. The corresponding pseudo-code is seen in Algorithm 7.

**Algorithm 7:** GPU step 3

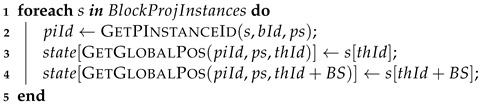



Auxiliary functions used on the GPU pseudo-codes are described in sequence:

- GetBlockId—provides the unique ID (identification) of the CUDA block;
- GetThreadId—gets the ID of the CUDA thread within the block;
- GetPInstanceId—obtain the ID for a given projection instance using the block ID and projection structure information;
- GetGlobalPos—obtain the position of amplitude in the global memory corresponding to a specific position in a projection instance.
- GetPInstancePair—provides a closed pair of positions in a gate projection structure for a given thread ID and a target qubit;
- MatchControls—verifies the control positions related to a quantum gate.

### 6.1. Main Results on CPU Evaluation Applications

The results reported in this paper consider that all tests were performed on a desktop with an Intel Core i9-7900X processor with ten cores, 32 GB RAM, and two NVidia GTX Titan X GPUs. The experiments were executed over Ubuntu Linux version 17.04, 64 bits, and CUDA Toolkit 9.0. Average simulation times were calculated from 30 executions, and all cases presented a standard deviation smaller than 1%.

Table 1 shows LIQUi|〉 and ProjectQ parallel performance results for simulations of Shor’s algorithm (on the left) and Grover’s algorithm (on the right). These results will be used later to make comparisons with the HybriD-GM results. Other simulators were not used since they were not available.

Now, we present the results achieved considering only CPU simulations. Shor’s and Grover’s algorithms were simulated sequentially and in parallel on CPU using the previous D-GM framework and the extension HybriD-GM, varying the number of threads from 1 to 10 and considering a range of qubits from 15 to 25.

The CPU layers perform the optimizations considering the cache size available in the target architecture; in the case of the simulations in this work (L2—1024 Kb, L1—32 Kb) the use of 16 qubits (512 kb) was defined for the size of the projection structure considering a coalescence of 11 qubits (16 Kb) in order to optimize the use of cache levels (avoiding full cache use).

#### 6.1.1. Shor’s Algorithm Simulations

Firstly, observe in Table 2 the execution times (in seconds) obtained for Shor’s algorithm simulations in the previous version of the D-GM framework.

**Figure 12:**
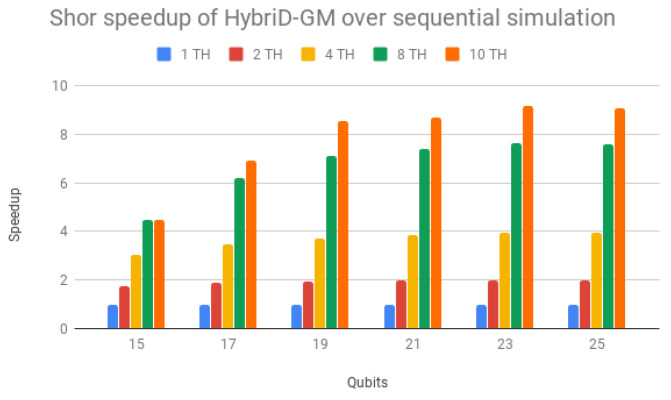
Shor’s algorithm speedups for HybriD-GM parallel simulation over sequential simulation.

Now, see Table 3 presenting the execution times (in seconds) for Shor’s algorithm in HybriD-GM. The speedups over the sequential simulation are shown in Figure 12. It is clear that all simulations with multiple threads showed performance gains, presenting better scalability for simulations with a higher number of qubits reaching 9.18× for a 21 qubit simulation. This occurs because simulations with 15 and 17 qubits have a very low execution time due to the small size of the quantum state (256 and 1024 kilobytes). Thus, operations other than amplitude computations have an impact on the simulation total time.

**Figure 13:**
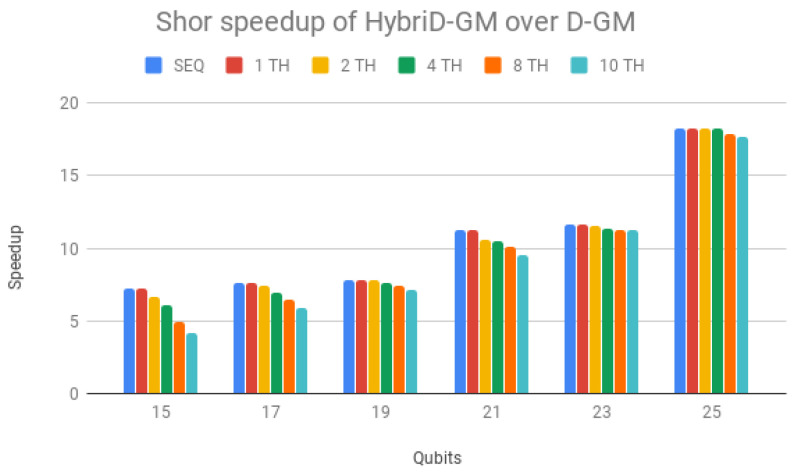
Shor’s algorithm speedup for HybriD-GM over D-GM.

HybriD-GM speedup over D-GM framework is presented in Figure 13, showing superior performance for all simulations, increasing with the number of qubits and achieving an 18× faster performance for 25 qubits simulations. Note that the speedup of the simulations in the HybriD-GM model considers the same execution scenario in relation to the D-GM model, that is, sequential simulations are compared with each other and the same is done to each number of threads in the parallel simulations.

Table 3 and Figure 12 show the simulation times and the speedup obtained in the HybriD-GM model with the increase in the number of threads, and it can be observed that for simulations with few qubits, the speedup presented does not approach the ideal. This occurs because the overhead caused by non-parallel computations, such as synchronization, has a significant impact on the total running time in such fast simulations. However, HybriD-GM’s speedups approach the ideal and stabilize with the increase in the number of qubits, when the impact of overheads becomes less significant.

These overheads do not impact so much on the D-GM performance because it has longer execution times, and therefore, in Figure 13, the simulations with few qubits show a smaller gain with the increase in the number of threads. The increase in the number of qubits yields speedups not only stabilizing for the different execution scenarios but also presenting greater scalability.

**Figure 14:**
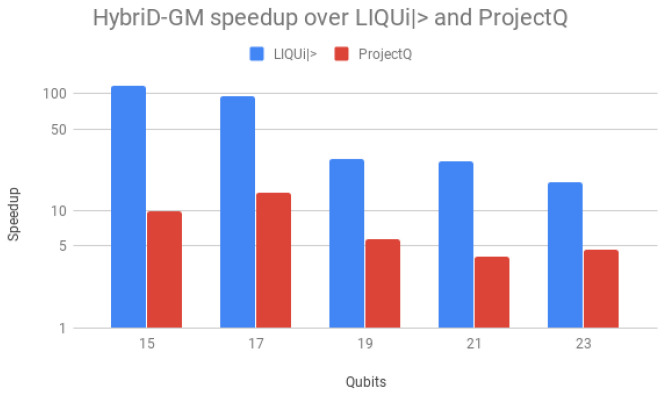
Shor’s algorithm speedup for HybriD-GM with 10 threads over LIQUi|〉 and ProjectQ.

Speedups over LIQUi|〉 and ProjectQ are shown in Figure 14, using a logarithmic scale for better visualization. Speedups over LIQUi|〉 started high, 117× for 15 qubits, but declined and stabilized with the increase in the number of qubits, down to 17.7×. For ProjectQ, the speedup presented a similar behavior but on a small scale, with a maximum of 14.2× with 17 qubits. HybriD-GM presented a better performance for all qubit values, achieving higher values for low qubits as a consequence of being more efficient than those simulators for these executions.

#### 6.1.2. Grover’s Algorithm Simulations

**Figure 15:**
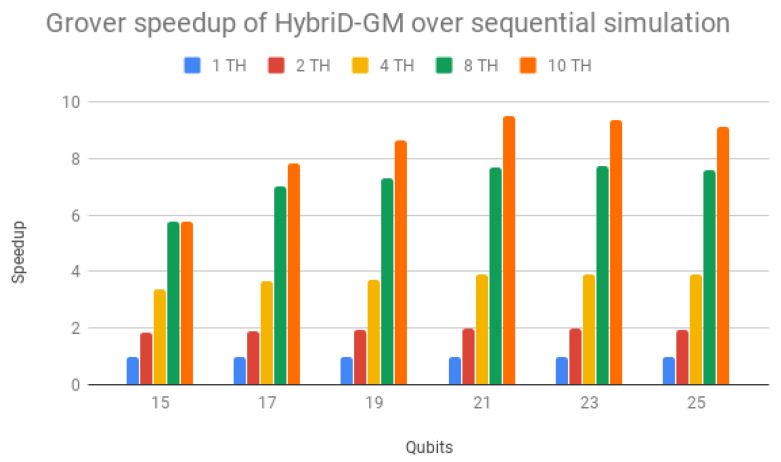
Grover’s algorithm speedups for HybriD-GM parallel simulation over sequential simulation.

By contrast, Table 4 shows the execution times obtained for Grover’s algorithm simulations in the D-GM framework, and Table 5 shows the execution times for CPU simulations of Grover’s algorithm in HybriD-GM. In Figure 15, the speedups are presented in relation to sequential simulation. Simulations with multiple threads also showed performance gains for all cases with scalability similar to Shor’s algorithm, reaching up to 9.51× for 21 qubit simulation.

**Figure 16:**
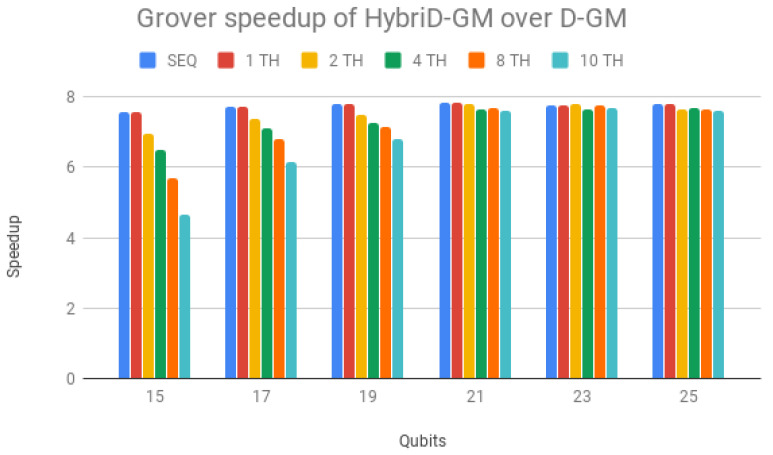
Grover’s algorithm speedup for HybriD-GM over D-GM.

HybriD-GM speedup over D-GM is seen in Figure 16, presenting steady scalability for 21-qubit simulations with a performance gain of around 7.9×. The same discussion provided for Figure 13 can be extended to the behavior shown in Figure 16.

One can notice that Shor’s algorithm presented a higher gain in performance. This happens because it benefited from improvements mostly acting on simulating controlled operators, and Grover’s simulation algorithm has less controlled operations.

**Figure 17:**
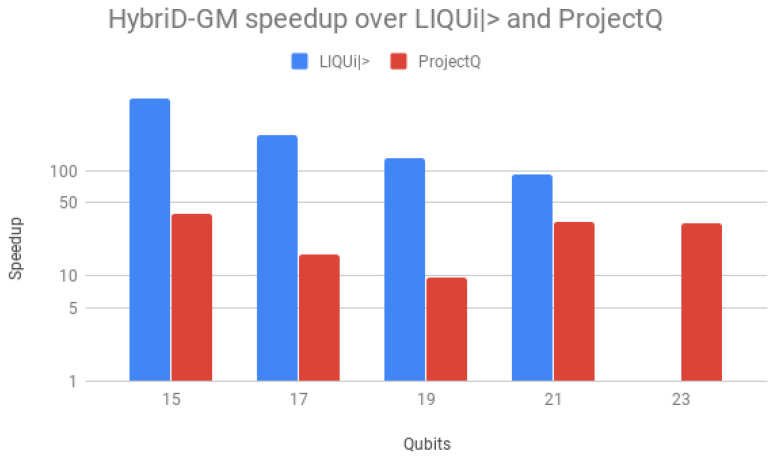
Grover’s algorithm speedup for HybriD-GM with 10 threads over LIQUi|〉 and ProjectQ.

Speedups over LIQUi|〉 and ProjectQ are shown in Figure 17, using a logarithmic scale for better visualization. Speedup over LIQUi|〉 also started high, 491.1× for 15 qubits, decaying and stabilizing as the number of qubits increased, down to 93.1×. For ProjectQ, the speedups went down from 39× with 15 qubits to 9.7× with 19 qubits, and then raised up to 32× for 21 and 23 qubits. This implies that ProjectQ loses performance in algorithms that have a small number of controlled gates. So, HybriD-GM presented an even better performance for Shor’s algorithm.

### 6.2. Main Results on GPU Evaluation Applications

In this section, the GPU evaluation applications are described, including the results achieved. We present the results obtained with isolated Hadamard gates. After, Shor’s and Grover’s algorithms are simulated and compared with the D-GM framework and our current HybriD-GM approach.

#### 6.2.1. Hadamard Quantum Transformation

We performed simulations containing a large amount of Hadamard gates (100,000) to understand the impact on the performance of coalescence and multiple operators per projection. This large number ensures that other factors, such as initialization and memory allocation, do not affect the total time.

The coalescing factor was varied from 0 to 6 (quantum state projection with $2^{0}$ to $2^{6}$ contiguous amplitudes) and the number of operators per each projection (i.e., per each kernel call) between 10, 50, 100, 150 and 200, for three execution types:

(1) *1 GPU*—using only one GPU;
(2) *2 GPU global*—using two GPUs and gates operating over the last qubit, which results in communication between GPUs when performing Steps 1 and 3 of the GPU kernel (detailed in Section 6);
(3) *2 GPU local*—using two GPUs and gates not operating over the last qubit, and, therefore, communication between GPUs is unnecessary.

**Figure 18:**
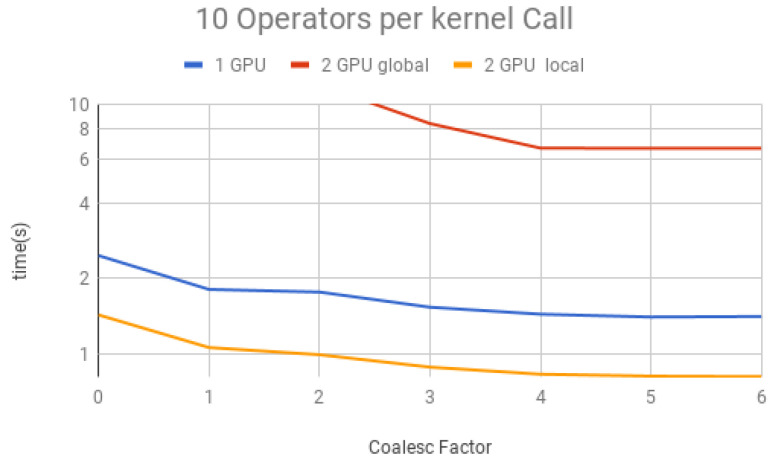
Projections—10 operators.

**Figure 19:**
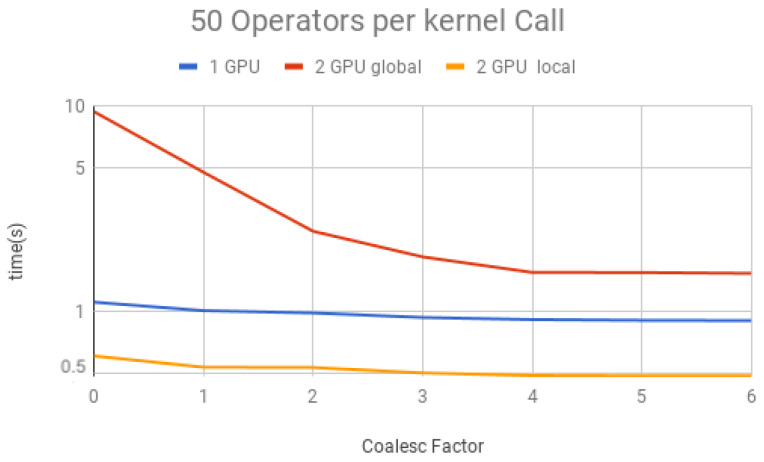
Projections—50 operators.

**Figure 20:**
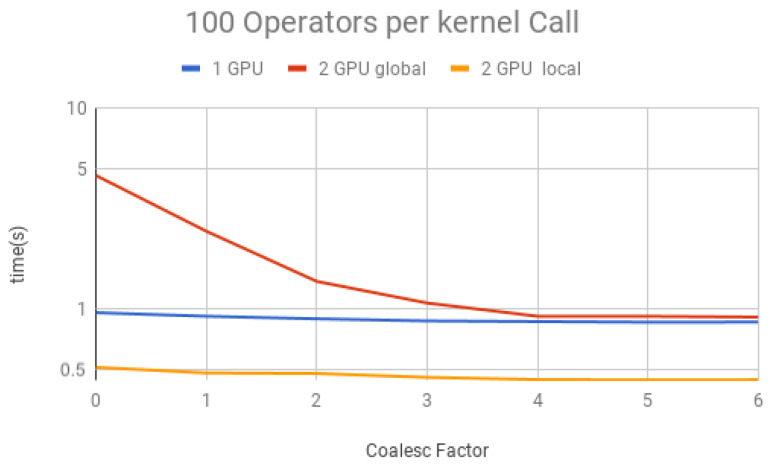
Projections—100 operators.

**Figure 21:**
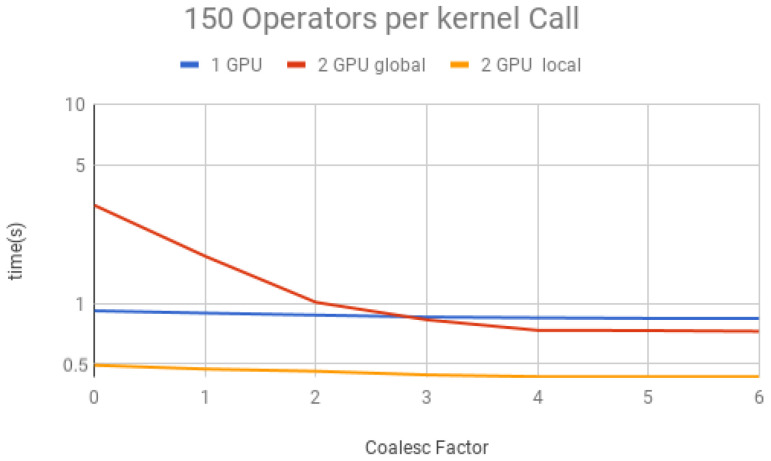
Projections—150 operators.

**Figure 22:**
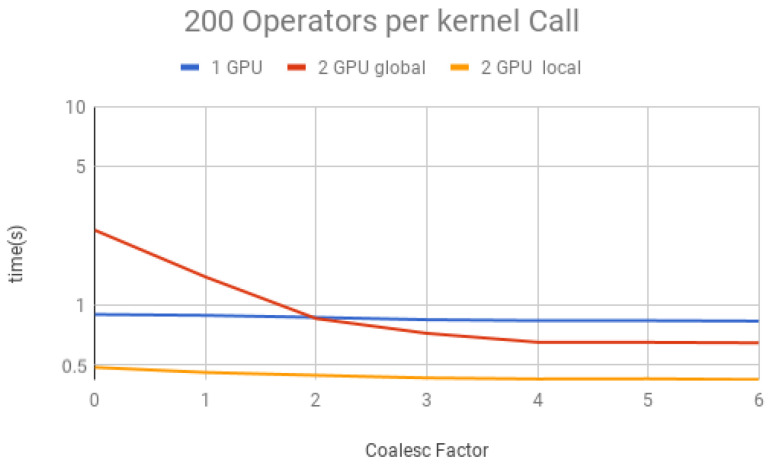
Projections—200 operators.

Simulation results for each structure described previously are presented in Figure 18, Figure 19, Figure 20, Figure 21 and Figure 22. Each figure presents the simulation time on the vertical axis, and the coalescing factor on the horizontal axis, increasing the number of gates per projection. Simulations using controlled gates would show similar results since only target qubits matter for the communication presented on Steps 1 and 3 of the GPU kernel.

Analyzing the results for the experiments seen in Figure 18 for 10 operators, Figure 19 for 50 operators, Figure 20 for 100 operators, Figure 21 for 150 operators, and Figure 22 for 200 operators, we can conclude the following:

(1) For all projections, increasing the coalescing factor up to four yields the simulation time decrease, having no fluctuations after that. This factor 4 determines that projection instances have $2^{4}$ coalesced amplitudes, resulting in a coalesced memory access of $2^{8}$ bytes (8 bytes per amplitude), which is the minimum size per memory request in this hardware, for its own and to another GPU within a computation node. Thus, any factor smaller than four will produce less efficient memory requests. Larger ones will be broken into multiple requests.
(2) Two GPU local was ≈2× faster than one GPU; this was expected since it has double the computational power and no communication between GPUs is required;
(3) Two GPU global is slower than one GPU for a small number of operators per projection because the communication between GPUs has a high cost. For 150 operators per call and coalesced factor 4, it was ≈1.3× faster, showing that as long as the time spent computing operators (Step 2 of the GPU kernel) is high enough, it can absorb some of the overhead inserted by the communication between GPUs, also achieving some improvement in performance for this type of execution.

#### 6.2.2. Shor’s Algorithm Application

**Figure 23:**
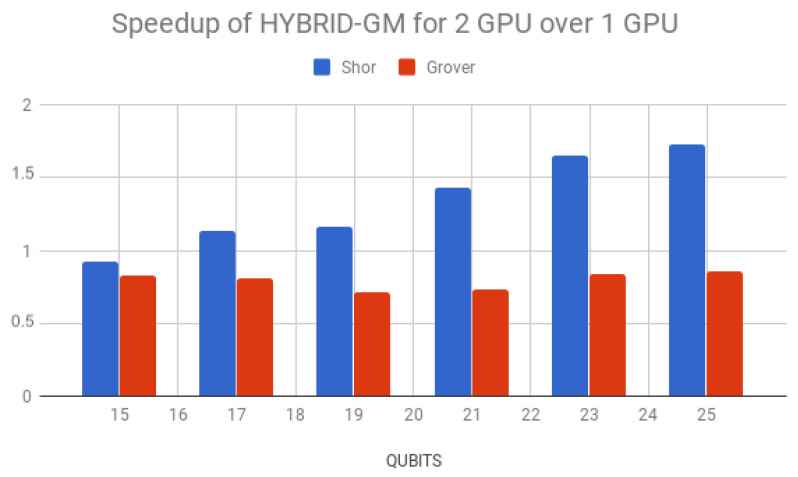
Speedup of simulations with 2 GPUs over 1 GPU.

Simulation times for Shor’s algorithm (in seconds) over the D-GM framework and HybriD-GM are presented in Table 6. HybriD-GM speedups over D-GM are shown in Figure 23 showing improvements for 2 GPUs over 1 GPU.

Regarding multi-GPU simulations on the HybriD-GM model, executions with two GPUs did not show performance gain for a small number of qubits due to the few computations. However, from 19 qubits forth, it showed growing speedups up to 1.73×.

#### 6.2.3. Grover’s Algorithm Application

**Figure 24:**
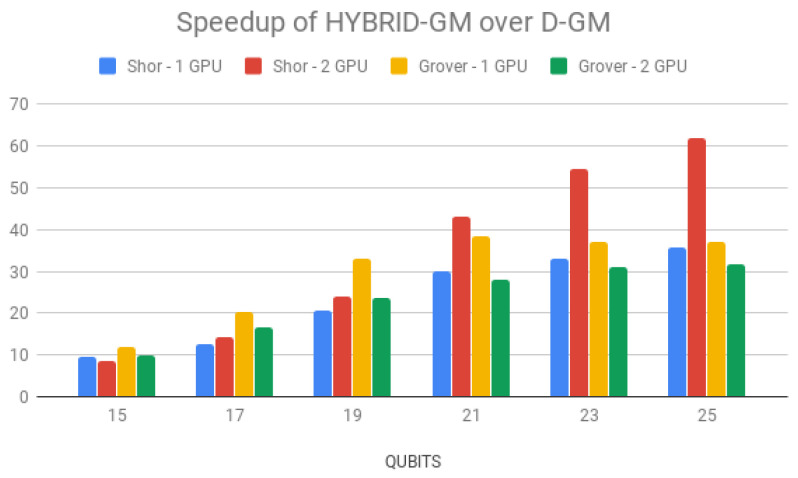
HybriD-GM speedup over D-GM for GPU simulations.

Simulation time for Grover’s algorithm using HybriD-GM and D-GM can be seen in Table 7. HybriD-GM speedups over D-GM are shown in Figure 24, also showing improvements up to 38.32× and 31.76× faster, for 1 and 2 GPUs, respectively.

Regarding multi GPU simulation performance, it achieved better speedups than Shor’s algorithm for all numbers of qubits, when using one GPU. This happens because the circuit for Grover’s algorithm has a lower average number of operators per projection, consequently making this application more constrained, and thus, the memory coalescing access feature has a bigger impact on the performance. For that same reason, the execution with two GPUs was slower than with a single GPU. Despite having coalesced access, communication between GPUs is slow and ends up having a large impact on runtime.

### 6.3. Exploring the Potential of HybriD-GM Model to Simulate Applications on the Extension of the D-GM Framework

In this subsection, we address the results when performing a hybrid simulation in CPU and GPU. For that, in the next simulations, the GPU memory is limited to represent a scenario where the GPU is not able to store the entire quantum state as occurred in the previous GPU simulations.

Simulations of Shor’s and Grover’s algorithm were performed for a 25-qubit simulation, limiting the GPU memory to 20 qubits, and the results are shown in Table 8. One can observe that the simulations using only GPU increased in time for both algorithms, in contrast to their times with no memory limitation. Shor’s algorithm only increased 1.31×, while Grover’s algorithm increased 8.91×. This happens because Shor’s algorithm can execute more operators per projection than Grover’s algorithm, and therefore it needs to perform fewer data transfers between CPU and GPU.

We realize that the hybrid simulation for Shor’s algorithm decreased the performance (in seconds) with the increase in the number of threads once the GPU had to wait for the threads to end their computation and go to the next projection. Grover’s algorithm presented an execution 1.22× slower with 1 thread, but the performance improved with the increase in the number of threads up to 8 threads, being up to 1.48× faster than the execution using only GPU. This happened because the computation performed by the threads was able to compensate for the idle time of the GPU since fewer operators are calculated per projection; the GPU does not have to wait as long for the threads to finish their calculations. In both algorithms, it was possible to observe a decrease when using the same number of threads as cores, and this happens because one thread is for controlling the GPU communications and, therefore, it will not be fully dedicated to the CPU computations.

The results showed that, depending on the algorithm, it is possible to gain performance using a hybrid approach when the quantum state cannot be fully stored in the GPU. Hybrid simulations on architectures that have a real limitation on GPU memory would likely benefit most from this approach.

## 7. Final Remarks

The simulation of QC can be developed as an important application for HPC, as spatial and temporal complexity increases exponentially in qubit dimensions of simulations. So, this work contributed to the development of the HybriD-GM model for quantum computing simulation. The model explored projection operators acting on quantum structures, such as QS and QT, manipulating not only coalesced memory data but mainly the granularity and distribution of quantum computations.

The HybriD-GM strategy for computation performances is modeled as a tree data structure, where intermediate and final nodes represent projection and execution layers, respectively. Such a structure is configured to optimize the hardware resources for some scenarios, which at the current state of the model are based on simulations in CPU, GPU, and hybrid approaches. The proposed optimizations discussed projection operators and their relationship in terms of tensor product decomposition related to QT and QS.

We performed a validation and evaluation of the HybriD-GM model by extending the D-GM environment so that the simulations occurred in the form established by the model. The D-GM framework extension considers the following:

- Addition of structural and functional modules providing the corresponding representation and manipulation of projection operators;
- Reorganization of CPU/GPU structures, supporting the HybriD-GM architecture;
- Implementation of the hybrid simulation, combining CPU/GPU approaches.

In this context, Table 9 reports an overview of the HybriD-GM approach and the simulators described in Section 2. It shows the main optimizations for QC simulation over multi-core CPU, multi-node CPU, and single-node GPU (with single or multiple GPUs). None of the simulators has a hybrid approach combining CPU and GPU, and despite having interesting optimization strategies, many of those simulators delve into a specific architecture. To tackle that, the proposal of our work considers a computational model for QC simulation that can either be applied to any architecture or optimize for its resources.

Simulations testing the HybriD-GM model’s efficiency were performed, mainly taking Shor’s and Grover’s quantum algorithms. Then, the results obtained were compared with the previous version of the D-GM framework, and with LIQUi|〉 and ProjectQ simulators. We achieved a positive outcome on all types of simulations, consolidating the D-GM environment as a quantum computing simulator and validating the HybriD-GM for modeling hybrid computation.

Parallel results of Shor’s and Grover’s algorithms (1 to 10 threads) on HybriD-GM showed performance improvements for Shor’s algorithm up to 18× and for Grover’s algorithm up to 7.9× over the D-GM model. In comparison to LIQUi|〉 and ProjectQ, higher speedups were achieved for simulations with low qubits, up to 117× for Shor’s and 491× for Grover’s algorithm. We also had good speedups for simulations with more qubits, with 4.64× for Shor’s and 32× for Grover’s algorithm.

Moreover, the HybriD-GM model supports new extensions, allowing the addition of optimization steps, projection/computation scenarios, and other simulation types. Additionally, such a proposal of a hybrid software architecture for quantum computing is conceived independently of the hardware. The computations can be performed from regular desktops for sequential simulations of multi-qubit quantum applications to clusters with multiple GPUs.

For simulations over GPU, the results proved that setting the projection coalescence to match the GPU memory requests size will improve performance, as contemplated by the HybriD-GM model. When compared to the previous D-GM version, simulations of Shor’s algorithm showed gains up to 35.74× and of Grover’s algorithm up to 38.32×. For simulations with 2 GPUs, only Shor’s algorithms were able to show improvements, up to 1.73×, as it presents a low amount of operators acting on the qubit that implies the necessity of communication between GPUs.

Further work in the HybriD-GM project consists of many research topics, including but not restricted to the following items: (i) reinforce the HybriD-GM model, (ii) improve memory access and memory use in QA simulation, and (iii) consolidate the simulation of fuzzy algorithms based on quantum computing.

## Figures and Tables

**Table 1:** Shor’s and Grover’s algorithm simulation times, in seconds.

	*Shor’s Algorithm*	
***Qubits***	**LIQUi|〉**	***ProjectQ***
15	16.90	1.43
17	54.91	8.15
19	77.22	16.00
21	430.39	66.27
23	2192.49	574.786
	***Grover’s Algorithm***	
***Qubits***	**LIQUi|〉**	***ProjectQ***
15	22.93	1.82
17	67.78	4.93
19	331.60	24.29
21	1879.58	656.16
23	−	5664.10

**Table 2:** Shor’s algorithm simulation over CPU using D-GM implementation.

*Qubits*	Seq.	1 Thread	2 Threads	4 Threads	8 Threads	10 Threads
15	4.66	4.66	2.44	1.29	0.70	0.59
17	30.24	30.24	15.65	7.96	4.14	3.38
19	186.81	186.81	96.52	48.98	24.85	20.06
21	1108.21	1108.21	573.53	290.98	157.48	132.35
23	13,223.30	13,223.30	6348.58	3276.57	1678.93	1134.27
25	35,278.31	35,278.27	18,142.40	9290.41	6403.29	6000.35

**Table 3:** Shor’s algorithm simulation over CPU for HybriD-GM, in seconds.

*Qubits*	Seq.	1 Thread	2 Threads	4 Threads	8 Threads	10 Threads
15	0.64	0.64	0.37	0.21	0.14	0.14
17	3.99	3.99	2.12	1.14	0.64	0.57
19	23.87	23.87	12.38	6.40	3.35	2.79
21	140.20	140.20	71.09	36.17	18.98	16.12
23	1138.27	1138.27	574.74	288.99	149.10	123.86
25	6320.18	6320.18	3167.27	1590.55	831.10	696.75

**Table 4:** Simulation times for Grover’s algorithm over CPU for D-GM, in seconds.

*Qubits*	Seq.	1 Thread	2 Threads	4 Threads	8 Threads	10 Threads
15	2.04	2.04	1.03	0.52	0.27	0.22
17	18.77	18.77	9.39	4.70	2.37	1.91
19	168.73	168.73	84.59	42.36	21.21	17.00
21	1504.03	1504.03	753.44	378.17	191.32	153.82
23	13,199.90	13,199.90	6619.73	3324.60	1701.69	1391.06
25	115,317.00	115,317.00	57,759.60	28,991.90	14,820.80	12288.80

**Table 5:** Simulation times for Grover’s algorithm over CPU for HybriD-GM, in seconds.

*Qubits*	Seq.	1 Thread	2 Threads	4 Threads	8 Threads	10 Threads
15	0.27	0.27	0.15	0.08	0.05	0.05
17	2.43	2.43	1.27	0.66	0.35	0.31
19	21.68	21.68	11.28	5.82	2.97	2.50
21	192.03	192.03	96.52	49.40	24.92	20.19
23	1696.62	1696.62	850.74	434.20	219.12	180.72
25	14,778.00	14,778.00	7534.92	3780.28	1942.71	1616.72

**Table 6:** Average simulation times for Shor’s algorithm (in seconds).

		*HybriD-GM*	
***Qubits***	***D-GM***	***1 GPU***	***2 GPUs***
15	0.76	0.081	0.088
17	2.48	0.198	0.174
19	11.83	0.571	0.492
21	63.84	2.116	1.480
23	363.39	11.01	6.648
25	2015.53	56.38	32.56

**Table 7:** Average simulation times for Grover’s algorithm (in seconds).

		*HybriD-GM *	
***Qubits***	***D-GM***	***1 GPU***	***2 GPUs***
15	0.19	0.015	0.019
17	1.04	0.051	0.063
19	6.97	0.210	0.0294
21	56.31	1.469	2.015
23	489.52	13.215	15.770
25	4245.33	114.723	133.629

**Table 8:** Simulation for a hybrid execution of 25-qubit Shor’s and Grover’s algorithms, limited by 20 qubits for the GPU memory.

	Only GPU	1 Thread	2 Threads	4 Threads	8 Threads	10 Threads
Shor	74.40	137.09	136.63	140.91	157.81	171.69
Grover	1023.20	1247.79	1202.79	1106.32	690.52	848.71

**Table 9:** Summary analysis of the literature review related to HybriD-GM and selected QC simulators.

		HybriD-GM	S1	S2	S3	S4	S5	S6
Multi-core	Model for							
	Parallel Programming	⋄	F#	⋄	⋄	⋄	–	–
	AVX support	**✗**	**✗**	**✗**	**•**	**•**	–	–
	Cache optimizations	**✗**	**•**	**•**	**✗**	**•**	–	–
	Instruction Reordering	**✗**	**✗**	**✗**	**✗**	**•**	–	–
Single- or Multi-GPU	Global Memory							
	Coalesced Access	**✗**	–	–	–	–	**•**	**•**
	Uses Shared Memory	**•**	–	–	–	–	**•**	**•**
	Uses Constant Memory	**•**	–	–	–	–	**✗**	**✗**
	Many gates							
	per kernel call	**•**	–	–	–	–	**•**	**•**
Distributed	With	**✗**	Azure	No API	**✗**	MPI	**✗**	**✗**
	Global Gates opt.							
	(controlled and sparses)	–	**✗**	**✗**	–	**•**	–	–
Circuit Opt.	Scheduling	**✗**	**✗**	**✗**	**✗**	**•**	**✗**	**✗**
	Gate Growth	**✗**	**•**	**✗**	**✗**	**•**	**✗**	**✗**

**•** implemented; **✗** not implemented; – cannot be implemented; ⋄ OpenMP.

## Data Availability

The data presented in this study are openly available in Github at https://github.com/abdavila/D-GM-QC-Simulator (accessed on 8 March 2023), and https://github.com/qflex-project (accessed on 8 March 2023).

## Abbreviations

The following abbreviations are used in this manuscript:

API	Application Programming Interface
CPU	Central processing unit
CUDA	Compute Unified Device Architecture
GPU	Graphics processing unit
HPC	High performance computing
MPI	Message Passing Interface
NL	Next layer
OpenMP	Open Multi-Processing
PL	Projection layer
QA	Quantum algorithm
QC	Quantum computing
QFT	Quantum Fourier Transformation
QS	Quantum states
QT	Quantum transformations
